# Plasma HER2ECD a promising test for patient prognosis and prediction of response in HER2 positive breast cancer: results of a randomized study - SAKK 22/99

**DOI:** 10.1186/s12885-020-6594-0

**Published:** 2020-02-11

**Authors:** Serenella Eppenberger-Castori, Dirk Klingbiel, Thomas Ruhstaller, Daniel Dietrich, Daniel Alexander Rufle, Karin Rothgiesser, Olivia Pagani, Beat Thürlimann

**Affiliations:** 1 University Hospital Basel, Institute of Human Genetic and Pathology, Schönbeinstrasse 40, CH-4031 Basel, Switzerland; 2 SAKK Swiss Group for Clinical Cancer Research Coordinating Centre, Bern, Switzerland; 3 F. Hoffmann-La Roche Ltd, Basel, Switzerland; 4 Brustzentrum Ostschweiz and University of Basel, St. Gallen, Switzerland; 5 University of Basel, Basel, Switzerland; 6 Breast Unit and Institute of Oncology of Southern Switzerland, Geneva University Hospitals, Swiss Group for Clinical Cancer Research (SAKK), Lugano, Viganello, Switzerland; 7 Breast Centre St, Gallen, Switzerland

**Keywords:** HER2+ breast cancer, Plasma HER2_ECD_ levels, Baseline, Trastuzumab, Sequential therapy, Upfront combined therapy

## Abstract

**Background:**

The HER2 extracellular domain shed in blood (HER2_ECD_) is reported to rise and fall in parallel with HER2+ breast cancer behavior. In this study, we evaluated the clinical relevance of plasma HER2_ECD_ values in patients with metastatic breast cancer treated in the SAKK22/99 trial comparing trastuzumab monotherapy followed by trastuzumab-chemotherapy combination at progression versus upfront combination therapy.

**Methods:**

Quantitative assessment of plasma HER2_ECD_ was performed in 133 patients at baseline; after 2–24 h; at 3 weeks; at first response evaluation (8–9 weeks); and at tumor progression. Associations with tumor characteristics, disease course and trial treatment were evaluated.

**Results:**

Baseline HER2_ECD_ levels were stable within 24 h after the first trastuzumab injection. These plasma values correlated positively with the HER2 gene ratio (r_s_ = 0.39, *P* < 0.001) and HER2 protein expression levels (r_s_ = 0.36, *P* < 0.001) but not with ER/PR status of the primary tumor. HER2_ECD_ baseline levels were positively associated with the presence of visceral disease (*P* = 0.05) and poor patients’ outcome (Cox-regression: *P* = 0.009). Patients with high baseline levels (> 35 ng/ml) had the worst overall survival (*P* = 0.03) if treated with upfront combination therapy. Conversely, patients with low HER2_ECD_ baseline values (< 15 ng/ml) had longer time to progression on combined trastuzumab-chemotherapy when first treated with trastuzumab monotherapy (*P* = 0.02). Monitoring HER2_ECD_ levels during the course of the trial revealed significant time (*P* = 0.001) and time-treatment arm interactions (*P* = 0.0007). Under upfront trastuzumab alone, the HER2_ECD_ levels remained stable until just before disease progression. In patients responding to combination treatment HER2_ECD_ levels decreased to > 20%.

**Conclusions:**

Plasma HER2_ECD_ levels in patients with metastatic breast cancer reflect HER2 disease status. This robust biomarker might help identifying patients without visceral disease profiting from a sequential treatment’s modality. Monitoring HER2_ECD_ levels during trastuzumab monotherapy could help defining the optimal time to introduce chemotherapy.

**Trial registration:**

Registration Number by ClinicalTrials.gov: NCT00004935, Trial number: SAKK22/99. Registered on 27 January 2003.

## Background

Epidermal growth factor receptor-2 (*HER2*) is an oncogene of key importance in breast cancer (BC). Its amplification occurs in 15–25% of primary BC patients and is age-dependent [1, 2]. HER2 amplification identifies an intrinsic subtype [3] of particularly aggressive BC correlating with higher levels of proteases [4]. HER2 represents the target of monoclonal antibodies, such as trastuzumab (T), pertuzumab and *T*-*DM1*, effective in the cure of patients with HER2 positive BC [5, 6] as well as of tyrosine kinase inhibitors (TKIs) [7, 8]. This protein located in the cytosolic membrane is composed of three domains: the internal tyrosine kinase reactive part, the transmembrane lipophilic linker and the extracellular carboxyl-terminal tail called extracellular domain (ECD) [9]. Metalloproteases can cleave the ECD from the cell surface. The HER2_ECD_ with a length of p105 represents the shed ECD product, which is circulating in blood of patients with BC cells overexpressing this oncogene and high levels of proteases. An enzyme-linked immunosorbent assay has been developed to dose HER2_ECD_ blood levels and proposed as peripheral marker for monitoring disease progression and predict therapy response [10–12]. This is of clinical interest since treatment of advanced BC is historically based on the HER2 status of the primary tumor and several studies have suggested a possible clinically significant discordance of up to 42% between the HER2 status at primary and later disease stages [13, 14]. The discrepant HER2 status during the course of the disease is however reported to be minimal (2%) when based on in situ hybridization detections and performed with exactly the same methods in a single center [15].

The HER2_ECD_ is detectable in serum and plasma, but the majority of studies analyze serum samples. The HER2_ECD_ test is Food & Drug Administration (FDA) approved and numerous clinical studies have demonstrated that both a HER2_ECD_ serum level > 15 ng/ml and a reduction of ≥20% between 2 successive blood draws is predictive for significant trastuzumab response [16].

The present study was designed as translational research investigation of the SAKK22/99 clinical study [17] to investigate associations of plasma baseline HER2_ECD_ values and longitudinal time points’ variations with treatment efficacy to possibly improve identification of patients requiring upfront combination of trastuzumab with chemotherapy (TChemo).

## Methods

### Inclusion criteria for the translational sub-protocol and ethical considerations

In short, women with histologically proven HER2-positive advanced BC without previous trastuzumab treatment or brain/meningeal involvement or concomitant serious diseases were randomly assigned (1:1) to trastuzumab alone followed, at progressive disease (PD), by combination with chemotherapy (Arm A) versus the upfront TChemo (Arm B).

The primary endpoint of this superiority trial was time to progression (TTP) on combined trastuzumab-chemotherapy (TTP-TChemo) in both arms. Secondary endpoints included response rate, TTP, overall survival, quality of life and toxicity. The T loading dose of 4 mg/kg intravenous (iv) was followed by 2 mg/kg iv weekly. In the initial 1st-line population (*n* = 84), chemotherapy was weekly paclitaxel 90 mg/m2 iv (3/4 weeks). After the amendment, chemotherapy was at the investigator’s choice (taxanes, vinorelbine, cisplatin) according to label indications/schedules. Chemotherapy could be stopped after ≥6 cycles in responding patients or after unacceptable toxicity, trastuzumab was continued until progression.

We collected and immediately centrifuged EDTA blood samples at baseline before the first trastuzumab infusion and 2–24 h afterwards and before chemotherapy in case of Arm B. Further plasma sampling occurred after 3 weeks, at first response evaluation (8–9 weeks), at tumor progression, and whenever feasible at later clinical assessments. The centers shipped plasma samples overnight at room temperature to a central laboratory in Basel. All patients signed a specific informed consent. Two patients withdrew their consent during the course of the investigation and we appropriately destroyed the correspondent plasma samples. Baseline HER2ECD values were obtained from 66 and 67 patients out of 87 and 88 patients enrolled in Arm A and B, respectively. Details of the clinical pathological issues of this subset of patients are summarized in Table 1.

**Table 1:** Clinicopathological patient’s characteristics

	Arm A (*n* = 66)	Arm B (*n* = 65)
Age (years)		
Median (range)	54 (33–78)	57 (33–72)
ECOG PS		
Unknown	1 (2%)	
0–1	65 (99%)	65 (100%)
N° CT regimens		
1st line	47 (71%)	46 (71%)
2nd-3rd line	19 (29%)	19 (29%)
ER status		
Positive	43 (65%)	38 (58%)
Negative	23 (35%)	27 (42%)
Adjuvant Anthracyclines		
No	39 (59%)	44 (68%)
Yes	27 (41%)	21 (32%)
Endocrine therapy		
No	14 (21%)	16 (24%)
Adjuvant	19 (29%)	24 (37%)
Palliative	8 (12%)	8 (12%)
Disease sites		
Bone only	6 (9%)	10 (15%)
Visceral only	20 (30%)	31 (48%)
Visceral + bone	24 (36%)	12 (18%)
Advanced disease at diagnosis (pM1)	21 (32%)	24 (37%)

### Enzyme-linked immunosorbent assay

Plasma HER2_ECD_ was quantified by means of a commercially available ELISA (Siemens Healthcare Diagnostics, Inc., Tarrytown, NY, USA) according to the manufacturers’ instructions. This is the same kit today approved by the FDA and available by Martell Diagnostic Laboratories, Roseville, MN, USA. Color intensity was measured on a COBAS EIA spectrophotometer (Hoffmann-La Roche Ltd., Basel, Switzerland). In general, samples were analyzed in batches every 6 months. Internal quality control was performed during each run using the controls provided with each kit. The results were expressed in nanogram per milliliter (ng/ml). Each sample, standard, and control was assayed in duplicate. Inter- and intra-assay coefficients of variation were less than 10%.

### Statistical analysis

HER2_ECD_ baseline levels just before and after trastuzumab infusion were compared to each other and to the primary tumor IHC HER2 scores, HER2 gene copy numbers, as well as oestrogen receptor (ER), progesterone receptor (PR) status using the Jonckheere-Terpstra and Wilcoxon rank sum test.

The test cut-off value recommended by the manufacturer in serum of primary and metastatic patients is 15 ng/ml [18–21]. This threshold is used to discriminate HER2+ from HER2 negative patients and is known to be indicative for bad prognosis under chemotherapy alone. Therefore, we analyzed the impact of this threshold value with respect to prediction to therapy response. Uni- and multivariate Cox-regression as well as the multitest function (Torsten Hothorn (2017) maxstat: Maximally Selected Rank Statistics. R package version 0.7–25. https://CRAN.R-project.org/package=maxstat) were used to investigate the clinical value of HER2_ECD_ levels with respect to time to progression (TTP) on combined trastuzumab-chemotherapy (TTP-TChemo) and to overall survival (OS).

Statistical analysis was performed with Statistical Package Software R (Version 3.4.1, 2017-06-30) www.r-project.org). *P* values < 0.05 were considered significant.

## Results

### Expression and association of baseline HER2_ECD_ levels with other makers

The HER2_ECD_ levels just before (*n* = 131) and after (*n* = 113) trastuzumab infusion varied within the normal repeated measurements variance independently from their levels. The detected ranges varied from 5 to 1427 ng/ml (median 26 ng/ml; mean 81 ng/ml) and 5-1543 ng/ml (median 27 ng/ml; mean 76 ng/ml) in samples before and after the first trastuzumab infusion with a resulting Pearson correlation coefficient of 0.995. This extremely high correlation is independent from the treatment arm (Arm A: *r* = 0.997; Arm B: *r* = 0.993). Subsequently, we considered the mean of these two detected values as baseline if both values (*n* = 113) were available. Otherwise, we took the single available value.

No difference was found between the baseline HER2_ECD_ levels of patients in Arm A and B, reflecting the random assignment of patients in the two arms.

**Fig. 1:**
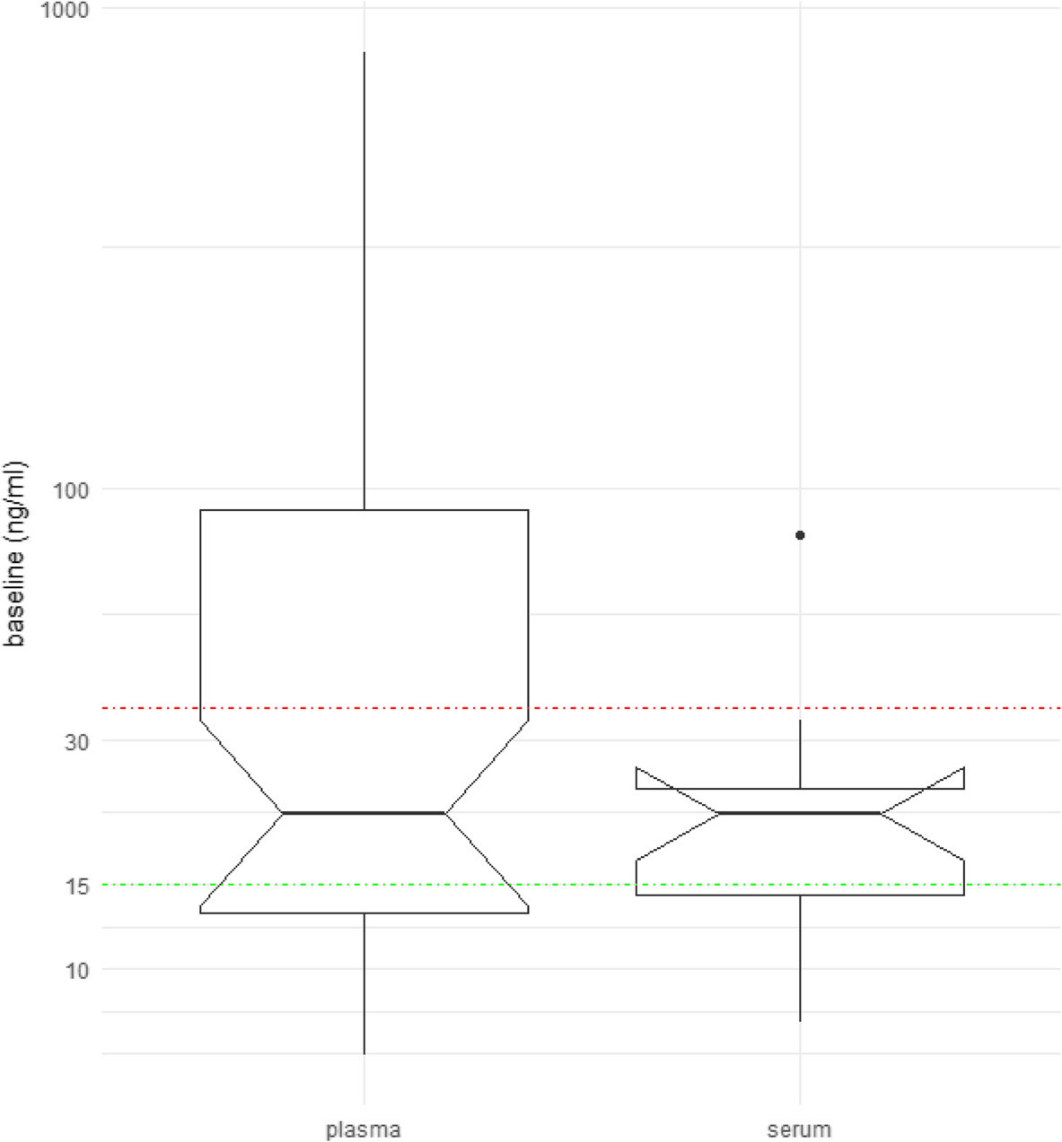
Comparison of baseline ECDHER2 values as detected in plasma (*n* = 47) or serum (*n* = 13) in two SAKK studies. Median values of plasma ECDHER2 levels of selected postmenopausal patients with ER positive tumors (left) compared to the one detected in serum of patients entering the SAKK23/04 (in both studies median: 21 ng/ml; *P* = 0.42)

Since the FDA approved threshold was based on serum detection, we compared the obtained plasma HER2_ECD_ data with the ones analyzed in serum of patients with similar age and menopausal status entering another trial (SAKK23/04; Fig. 1). The median value of both plasma and serum cohorts was 16 ng/ml, similar to the one reported by the literature for HER2+ BC (12 to 15 ng/ml) [22]. However, 40% of the HER2_ECD_ plasma values were above 30 ng/ml, which represents the maximal values detected in serum samples of the SAKK23/04 and literature.

These plasma baseline mean values correlated positively with the HER2 gene ratio (r_s_ = 0.39, *P* < 0.001) and HER2 protein expression levels (r_s_ = 0.36, *P* < 0.001). The HER2_ECD_ levels were found significantly (*P* < 0.001) higher in patients with HER2 Dako 3+ (median; range: 40; 6-1485 ng/ml; *n* = 83) as compared to Dako 2+ (13; 7-138 ng/ml; *n* = 29) as detected locally in primary tumors. No significant difference was found between the HER2_ECD_ baseline values and other available parameters such as ER and PR expression (*P* = 0.64 and *P* = 0.68), bone disease (*P* = 0.40) or number of metastatic sites (*P* = 0.37). Only a positive trend was observed between HER2_ECD_ levels and the presence of visceral disease (*P* = 0.05).

### Association of baseline HER2_ECD_ levels with therapy response and clinical outcome

**Fig. 2:**
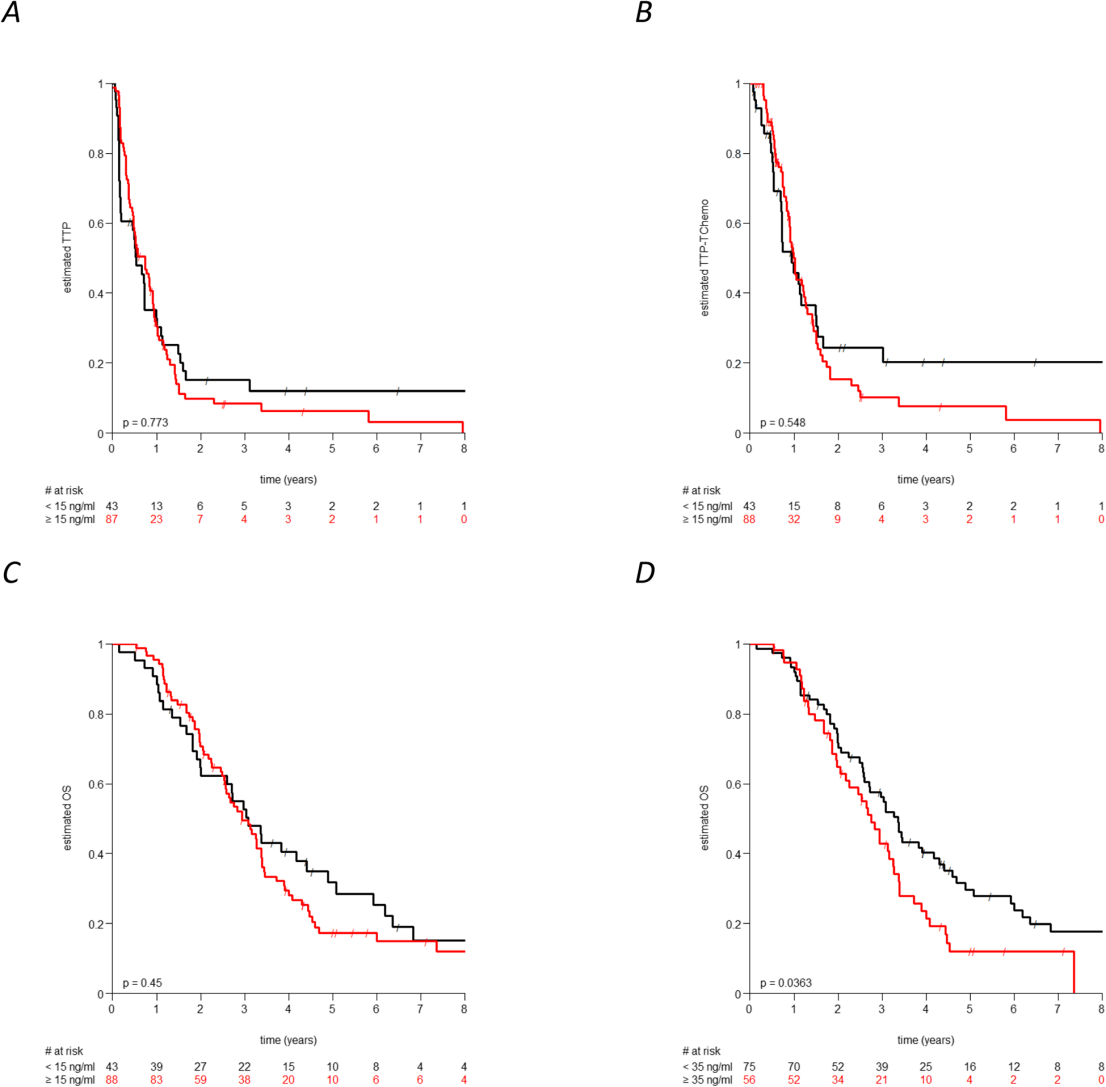
Kaplan–Meyer curves of the overall study population with respect to **a** TTP, **b** TTP-TChemo; **c** OS using the threshold of 15 ng/ml; and **d** OS using the calculated threshold of 35 ng/ml

Considering the threshold of 15 ng/ml, Kaplan-Maier curves and log-rank analyses of the overall population (i.e. Arm A and B) revealed no significant impact of baseline HER2_ECD_ plasma values on disease progression (TTP and TTP-TChemo) or OS (Fig. 2a-c).

However, univariate Cox-regression analysis revealed a significant (*P* = 0.009) association between the HER2_ECD_ levels, when analyzed as a continuous variable, with respect to OS. Moreover, in a multivariate Hazard Cox-regression analysis including baseline HER2_ECD_, ER- and PR-Status, treatment arm and the presence/absence of bone or visceral diseases, baseline HER2_ECD_ retained an independent significant prognostic factor (HR:1.2; CI:1.1–1.3 using logarithmic transformed data; *P* = 0.026) with respect to OS. In this model the presence of visceral disease was the only other significant parameter (HR:1.6; CI:1.3–2.2; *P* = 0.033).

Therefore, by means of *maxstat* functions we searched for the most appropriate threshold plasma value in our cohort of patients with advanced BC. As depicted in Fig. 2d, patients with baseline values ≥35 ng/ml had a significant (*P* = 0.04) worse OS compared to those with lower levels. After 4 years only 21.4% (CI:13–37%) of the patients with baseline HER2_ECD_ levels ≥35 ng/ml were still alive, while the OS rate was of 40% (CI:30–53%) for patients with lower HER2_ECD_ levels.

**Fig. 3:**
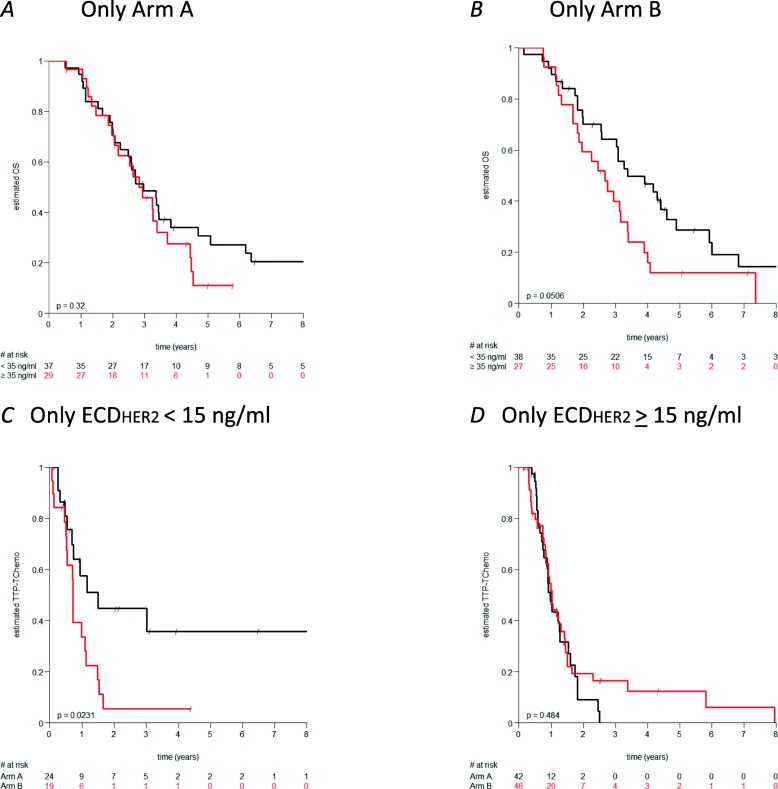
Kaplan–Meyer curves of patients’ subsets with respect to OS (**a*-*b**) and TTP-TChemo (**c**-**d***)*. Baseline HER2_ECD_ threshold values are 35 ng/ml in (**a**-**b**)*,* and 15 ng/ml in (**c**-**d**)

Interestingly, patients in Arm A treated upfront with trastuzumab alone showed no OS difference regardless of the baseline HER2_ECD_ level (Fig. 3a), in contrast to the whole population (Fig. 2d). Conversely, the observed good prognostic HER2_ECD_ impact on OS is consistent for patients treated with TChemo upfront (Fig. 3b).

Of particular relevance, patients with low levels of baseline HER2_ECD_ (< 15 ng/ml) have a significantly better TTP-Tchemo if first treated with T alone. One year after study treatment start, no progression is observed in 58% (CI:39–86%) of patients treated in Arm A, compared to 28% (CI:13–6%) for patients in Arm B (Fig. 3c).

### Monitoring HER2_ECD_ levels during trastuzumab and TChemo treatments

**Fig. 4:**
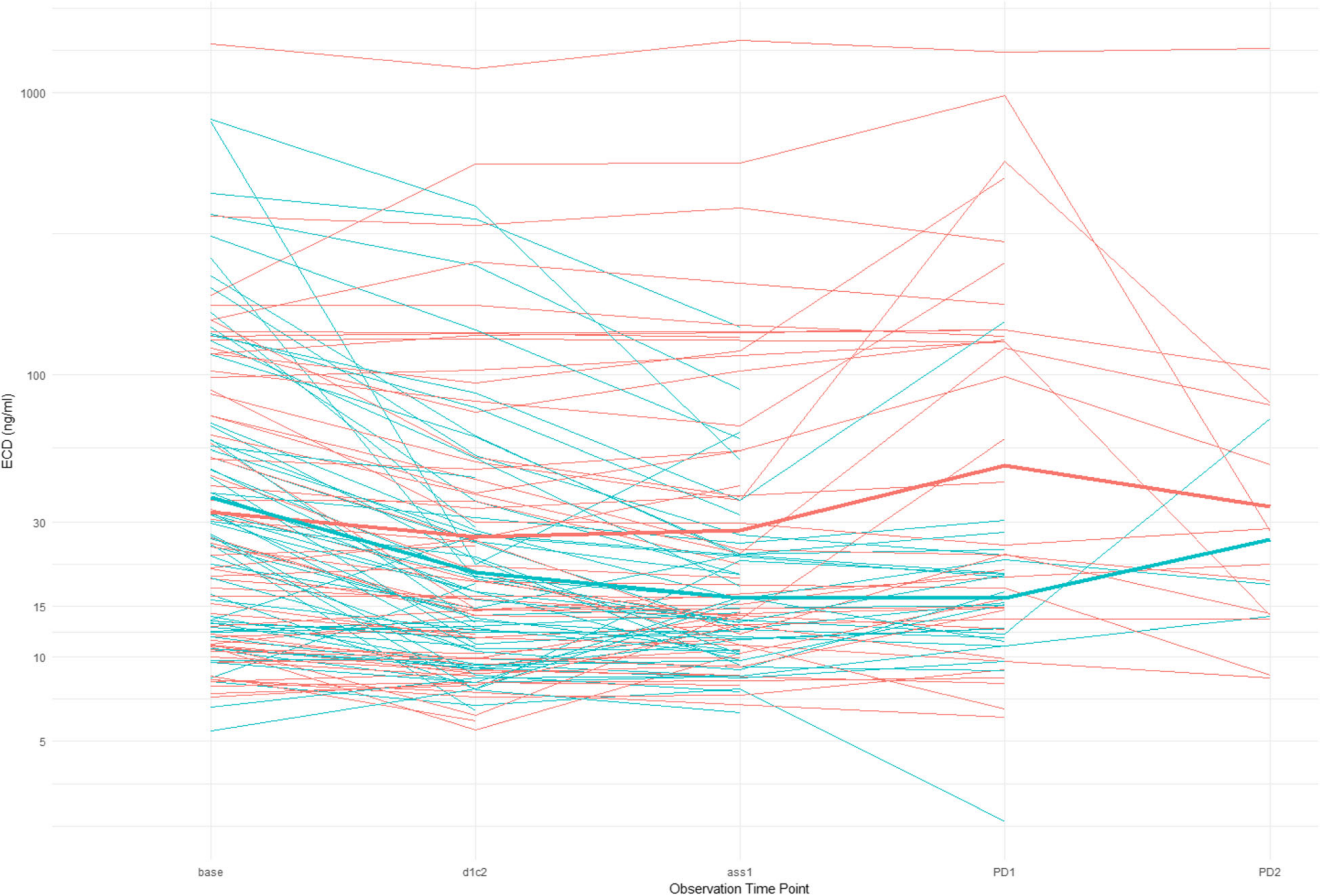
Spaghetti plots with summary of plasma HER2_ECD_ levels of the two arms’ cohort behavior: red lines Arm A and turquoise lines Arm B. Observation time points: Base: Baseline mean values before-after first injection; d1c2: day 1 s cycle (after 3 weeks); ass1: first assessment at 8–9 weeks; PD1: first progression; PD2: second progression

As shown in Fig. 4 and calculated by analysis of variance, we observed a significant time effect (*P* = 0.001) and time-treatment interaction (*P* = 0.0007). In case of therapy response, the HER2_ECD_ levels in patients receiving upfront TChemo (Arm B) decreased to a mean value of 15 ng/ml. This decrease represents > 20% of the respective baseline values. These low values persisted until disease progression and rose thereafter. In contrast, HER2_ECD_ levels did not change in patients responding at upfront trastuzumab alone (Arm A). Of interest, these values strongly increased (more than 20% of the respective baseline levels) at first disease progression (PD1).

**Fig. 5:**
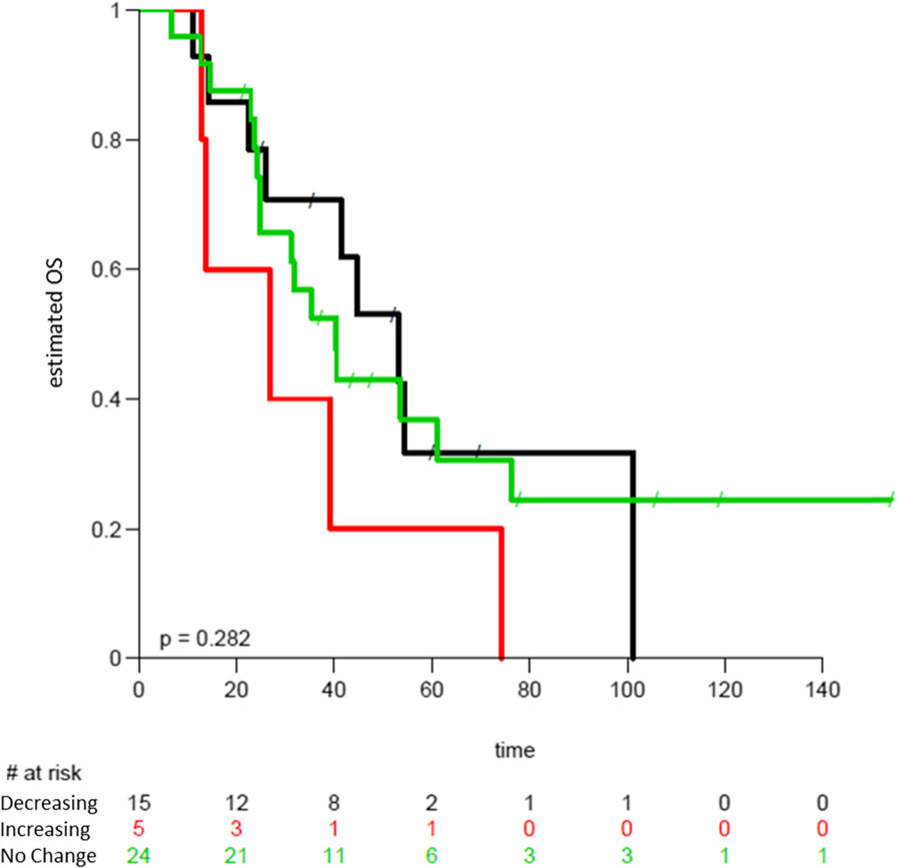
Kaplan-Meier curves depicting the OS of patients in Arm A with no change (green), increased (red), and decreased (black) ECDHER2 values at first assessment compared to baseline values

Lastly, we investigated if a decrease or increase within the first 9 weeks of at least 20% of HER2_ECD_ plasma levels with respect to the baseline values at first infusion could predict a longer TTP or OS. For patients treated upfront with trastuzumab alone (Arm A) there was a trend for better OS in case of quick decreased HER2_ECD_ levels versus worst OS in case of increased levels (Fig. 5).

## Discussion

Our translational research study aimed to address the medical need to identify a robust non-invasive marker able to select patients with particular profit from a sequential anti-HER2 chemotherapy treatment strategy. An approach which, according to our results of the randomized Phase III trial SAKK22/99, might help deferring chemotherapy and its toxicity fitting in a de-escalating intention in the treatment of HER2-positive disease [17].

Our results indeed indicate that patients with low baseline HER2_ECD_ levels may profit of single agent trastuzumab, while no difference between arms is observed when the baseline HER2_ECD_ levels are higher (Fig. 3c/d). Moreover, since the HER2_ECD_ levels under trastuzumab monotherapy remain fairly constant over time and start increasing only shortly before disease progression, monitoring HER2_ECD_ levels may help identifying the optimal moment for introducing chemotherapy.

To our best knowledge, this is the first study investigating plasma HER2_ECD_ or any other blood marker, including circulating tumor cells (CTCs) or free DNA, to address specifically the optimal time point for the introduction of chemotherapy after anti-HER2 monotherapy in HER2+ metastatic BC.

Several studies have investigated the value of serum HER2_ECD_ in patients with BC. Our results are in line with a recent review summarizing 27 studies (10 in metastatic BC, 13 in early BC and 4 in patients with early or metastatic BC) [18]. Even though there are discrepancies between studies, including the definition of the optimal threshold, the majority suggest that serum HER2_ECD_ might be particularly useful in metastatic BC as an indicator of cancer progression and predictor for anti-HER2 therapies efficacy.

The mechanistic reasons why higher levels of HER2_ECD_ could correlate with poorer prognosis and with less anti-HER2 therapeutic effectiveness are several. First of all, the truncated form that remains in the cancer cell membrane and cytoplasm after cleavage of the “shedding” ECD has a higher rate of constitutive tyrosine kinase activity and is therefore more oncogenic than the intact HER2 [19]. Second, HER2 is truncated and released into blood circulation by a proteolytic mechanism, which has been attributed to various zinc-containing metalloproteases including members of the matrix metalloproteinase and the ADAM family [20]. Subsequently, the presence of free HER2_ECD_ is also an indicator of presence in the cancer cells of proteins degrading the extracellular matrix and known per se as markers of invasiveness and aggressiveness. Third, if the external domain is missing the anti-HER2 therapy might bind the free HER2_ECD_ instead of the HER2 external domain lacking the targeted receptor blockage [18].

These mechanistic models along with our longitudinal monitoring of plasma HER2_ECD_ in the two treatment-arms help explaining our findings. Indeed, we observed longer TTP for patients with plasma HER2_ECD_ below 15 ng/ml only when treated with sequential treatment. Trastuzumab alone in these patients binds to the full-length HER2 domain and is effective. In this situation, there is no interaction between the free HER2_ECD_ and trastuzumab and the free HER2_ECD_ levels remain constant until the cancer progresses. At this point, the HER2_ECD_ levels increase and sequential HER2_ECD_ monitoring would allow identifying the exact moment for chemotherapy administration. According to protocol plasma collection timelines (baseline, after 3 weeks, at first response evaluation (8–9 weeks) and at PD) it was not possible to know how long before PD the HER2_ECD_ levels started to raise. Prospective studies should integrate monthly detections in order to estimate the possible anticipation of chemotherapy administration.

In contrast, we observed that HER2_ECD_ levels in patients treated with upfront TChemo, as reported in several other studies [21], continuously diminished, up to < 20% of the baseline level, when chemotherapy is effective. Consequently, patients with high levels of free ECD should be treated with upfront combination therapy.

Another recent meta-analysis based on 15 prospective and 8 retrospective studies, investigating the prognostic value of HER2_ECD_ with the FDA approved threshold of 15 ng/ml, concluded that higher levels are correlated with poorer OS with a hazard ratio (HR) of 2.3 (CI:2.0–2.6) [23]. Our results are very much in line with this meta-analysis despite our threshold, with respect to OS for patients treated with upfront TChemo, had to be set at a higher level (35 ng/ml).

One possible explanation for our higher threshold and overall expression values of the plasma HER2_ECD_ could be that the plasma test also detects the extracellular domain of HER2 present on CTCs. If this hypothesis is correct, plasma HER2_ECD_ could be more sensitive and representative of the aggressiveness of the disease and subsequently more clinically useful than the serum test, indicating the concomitant presence of cleaving proteases and CTCs facilitating metastatic processes.

Further, in this translational study, we confirmed that monitoring of HER2_ECD_ values represent a clinically relevant complementary assessment in order to compensate inter-laboratory and inter-observer discrepancies in HER2 overexpression, particularly when detected in small biopsies of metastatic disease or in very old tissue specimens.

Another relevant finding is the high correlation of plasma levels at baseline and within 24 h after trastuzumab infusion in both arms. This observation is clinically important since it supplies evidence for avoiding unnecessary double blood assessments and suggests that trastuzumab does not bind immediately to the free soluble HER2_ECD_.

This study was well conceived at time of trial start almost 20 years ago. Unfortunately, the trial recruited too slowly and did not achieve the planned number of patients. Therefore, the overall analysis is underpowered and the current treatment of choice pertuzumab/trastuzumab is investigated in a more recent study (SAKK22/10).

## Conclusion

In conclusion, our data suggest the detection of plasma HER2_ECD_ may help identifying patients with HER2+ BC who would profit from a sequential treatment and deciding when to introduce chemotherapy. According to our study and to the available literature serum or plasma HER2_ECD_ levels should enter routine clinical practice for monitoring metastatic HER2+ BC. Even though fine-tuning on the threshold is necessary, serum and plasma HER2_ECD_ might help to personalize anti-HER2 and chemotherapy regimens.

## Data Availability

All data are stored by the SAKK and rests of material is stored at the biobank of Pathology University hospital Basel and under the ownership of the SAKK. Data and rests of material are available from the SAKK on reasonable request.

## Abbreviations

BC: Breast cancer
CTC: Circulating tumor cells
ECD: Extracellular domain
ER: Estrogen receptor
FDA: Food & Drug Administration
HER2: Epidermal growth factor receptor-2
HER2_ECD_: HER2 extracellular domain
HR: Hazard ratio
iv: intravenous
OS: Overall survival
PD: Progressive disease
PR: Progesterone receptor
SAKK: Swiss Group for Clinical Cancer Research
T: Trastuzumab
TChemo: Trastuzumab with chemotherapy
TKI: Tyrosine kinase inhibitor
TTP: Time to progression
TTP-TChemo: TTP on combined trastuzumab-chemotherapy
